# “Three-dimensional evaluation of breast volume changes following autologous free flap breast reconstruction over six months”

**DOI:** 10.1016/j.breast.2020.02.005

**Published:** 2020-02-10

**Authors:** Floor N.H. Wilting, Marijn Hameeteman, Hanneke J.P. Tielemans, Dietmar J.O. Ulrich, Stefan Hummelink

**Affiliations:** Department of Plastic and Reconstructive Surgery, Radboud University Medical Center, Nijmegen, the Netherlands

**Keywords:** Autologous reconstruction, Breast reconstruction, Free flap, Volume change, 3D imaging, 3D stereophotogrammetry

## Abstract

**Objectives:**

To date, little is known about postoperative changes in breast volume after autologous breast reconstruction. The purpose of this retrospective study was to investigate breast volume changes following autologous free flap reconstruction and the factors affecting flap volume.

**Materials and methods:**

Patients who underwent deep inferior epigastric perforator, superficial inferior epigastric artery and profunda artery perforator flaps between December 2016 and January 2019 were included. Exclusion criteria were breast complications requiring surgical debridement, and the absence of at least two suitable three-dimensional images postoperatively. Three-dimensional stereophotogrammetry volume measurements were performed at the time of standard surgical check-ups. Changes in breast volume were modeled using a quartic polynomial curve function in a nested mixed effects model.

**Results:**

136 breasts in 101 patients were included. An average decrease of predicted breast volume was found from 637.8 cc (95%-CI [624.4, 651.1]) at two weeks to 566.6 cc (95%-CI [535.1, 598.0]) after three and 567.6 cc (95%-CI [515.9, 617.6]) after six months postoperatively. Reconstruction timing and first postoperatively measured breast volume showed a statistically significant difference in initial reconstructed breast volume and in the shape of the relationship between time and breast volume, whereas autologous technique and BMI only showed a statistically significant difference in initial reconstructed volume and mastectomy indication in the shape of the relationship.

**Conclusion:**

The final overall flap volume decreased to 88.9% of its original volume after six months. Gaining more insight into the factors influencing flap volume is of crucial importance to facilitate predictable surgical outcomes.

## 1 Introduction

Breast cancer is the most common cancer in women worldwide. Due to the improved survival rates of breast cancer over the past decades and the increasing trend in the use of bilateral and contralateral prophylactic mastectomy, more women continue to live with the consequences of mastectomy [[1], [2], [3], [4]]. Previous research has established that breast reconstruction supports the recovery of patients to a large extent by reducing psychological, social and sexual morbidity associated with the loss of the breast. The goal of breast reconstruction hereby is to recreate a breast that is naturally shaped, looks and feels like a normal breast, and ideally can mature and change with the patient over time. Autologous breast reconstruction is particularly appreciated for its longevity and for resulting in a typically more natural breast in appearance and feeling compared to implant-based breast reconstruction. Moreover, patients who undergo autologous reconstruction have greater satisfaction with their breasts and greater psychosocial and sexual well-being compared with patients who undergo implant reconstruction [5,6].

To achieve the desired breast size for autologous breast reconstruction, the required flap volume needs to be determined. In addition to harvesting the required flap volume, it is also important to know how much of the flap volume will ultimately remain. Although multiple studies have been conducted into the quantitative analysis of postoperative changes in flap volume in head and neck reconstruction, there is still a paucity of literature on postoperative flap volume changes following autologous free flap breast reconstruction [[7], [8], [9], [10]]. Three-dimensional (3D) stereophotogrammetry is a convenient measurement instrument for mapping these breast volumes, due to its fast capture speeds, ease of use and lack of discomfort and radiation exposure. 3D stereophotogrammetry is, however, hitherto mainly used during the preoperative planning in breast surgery, with only a few studies available of its use in the postoperative phase [[11], [12], [13]].

By providing data on postoperative breast volume changes, a prediction can be made of the expected postoperative course and final breast volume for each patient, allowing the surgeon to better predict the postoperative result in the future. This can be used to adjust the preoperative planning more precisely to the expectations and wishes of the patient and to prevent potential secondary correction procedures in terms of volume asymmetry. Therefore, the objective of this retrospective study was to investigate volume changes of autologous free perforator flaps used for reconstructive breast surgery utilizing 3D stereophotogrammetry and to clarify the factors that positively or negatively affect breast volume.

## 2 Methods and materials

All patients who underwent autologous free flap breast reconstruction in the Department of Plastic and Reconstructive Surgery at Radboud University Medical Centre between December 2016 and January 2019 were analyzed. Deep inferior epigastric perforator (DIEP), superficial inferior epigastric artery (SIEA) and profunda artery perforator (PAP) flaps were included. 3D images up to twelve months postoperatively were used for analysis. If no 3D image was available from the period of ten to twelve months postoperatively, the next captured 3D image was taken for analysis. 3D images were excluded for analysis for subsequent measurement moments after a volume-altering breast procedure (lipofilling, liposuction, reduction, mastopexy or flap mobilization) was performed on the reconstructed breast during the follow-up period. Exclusion criteria for enrollment were: total or partial flap loss and other breast complications requiring surgical debridement, and in the absence of at least two suitable 3D images postoperatively. The principles outlined in the Declaration of Helsinki have been followed and all study participants gave written informed consent.

Patient data were collected from the electronic medical records, including age, BMI, smoking status, mastectomy indication, type and timing of reconstruction, systemic (neo)adjuvant treatment, irradiation status, genetic mutations and ischemia time. In this study, primary breast reconstruction is defined as a reconstruction at the time of the mastectomy and secondary reconstruction when performed during a subsequent operation after the mastectomy, including patients who underwent delayed autologous reconstruction after immediate tissue expander placement. Ultimately, tertiary breast reconstruction is defined as a redo reconstruction in case of breast reconstruction failure or unsatisfactory results [14].

### 2.1 3D stereophotogrammetry and breast volume measurements

All 3D images were acquired with the VECTRA XT stereophotogrammetry system (Canfield Scientific, Inc., Parsippany, NJ, USA), in use at our outpatient clinic from April 2017. The 3D images were routinely acquired by a medical photographer at the time of the standard surgical check-ups around two weeks, three months, six months and twelve months postoperatively as part of our department’s standard imaging protocol. For logistical reasons, the timing of the appointments could deviate a few days for the two weeks, and a few weeks for the three, six and twelve months follow-up moments. The images were acquired with the patients in a standing position with arms in 30–45° abduction.

**Fig. 1:**
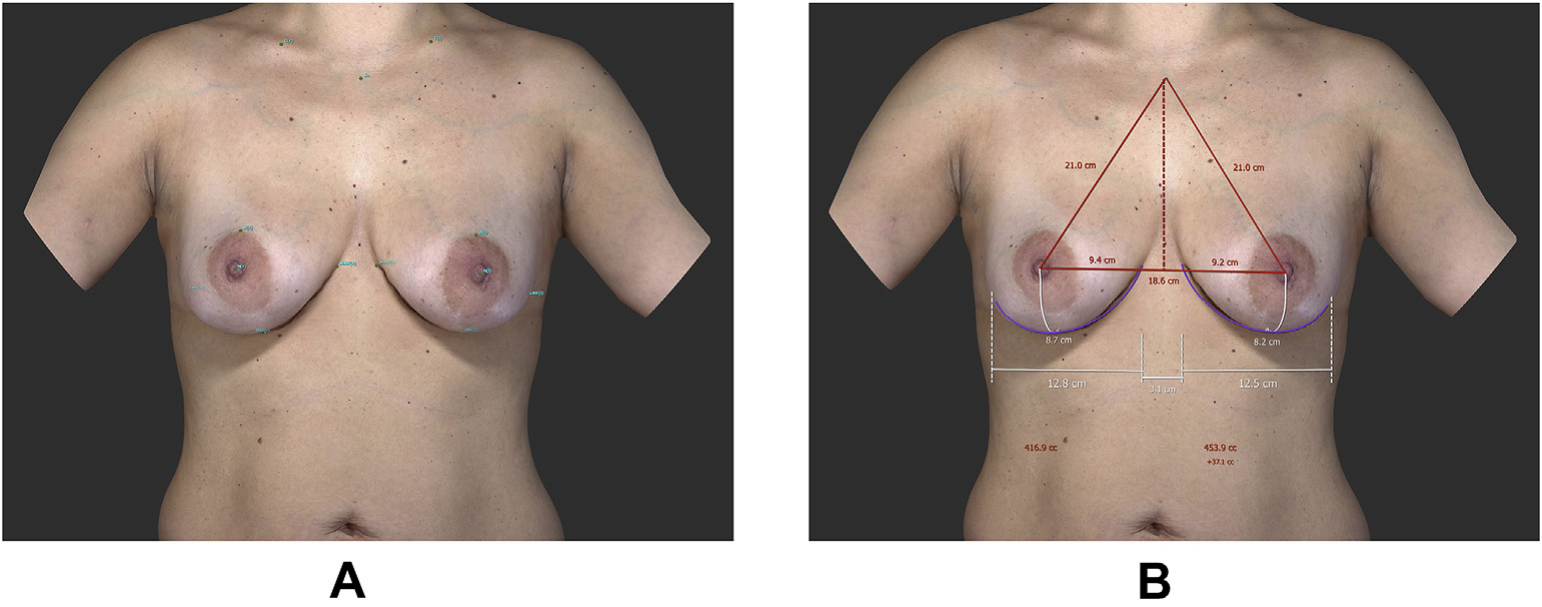
Breast volume measurement. (A) The boundaries of the breasts are selected, (B) The breast volume is measured.

To determine the breast boundaries, the following seven landmarks per breast were placed manually: sternal notch, midclavicular, nipple, areola border, inframammary fold and medial and lateral aspects of the mammary fold. Subsequently, breast volumes were calculated in cubic centimeters (cc) after a chest wall was simulated from the landmarks (Fig. 1). All measurements were performed by the same medical photographer using the built-in VECTRA software (version 5.8.6.).

### 2.2 Statistical analysis

The baseline characteristics were analyzed using descriptive and frequencies statistics in IBM SPSS Statistics, Version 25.0 (IBM Corp., Armonk, N.Y., USA). Differences in baseline characteristics between groups were compared using an independent samples *t*-test.

A nested mixed effects model was fitted to model the relationship between time after autologous breast reconstruction and breast volume. The data contained multiple time points across patients and, in case of bilateral reconstruction, data for each of two breasts per patient. The nested mixed effects model accounts for the correlation between breast volume across multiple time points and for the correlation between breasts for each individual. A fourth-degree polynomial transformation of time was fitted to model the relationship, as the relationship was seen to be non-linear. The time variables were standardized to reduce multicollinearity by subtracting the mean and then dividing by the standard deviation and were included in the model as fixed effects. To predict the population average of breast volume change over time, the following model was created: $y=\beta_{0}+\beta_{1}x+\beta_{2}x^{2}+\beta_{3}x^{3}+\beta_{4}x^{4}$ (model 1), where y is breast volume, $\beta_{0}$ is the intercept, $\beta_{1-4}$ are regression coefficients and x is the standardized time postoperatively.

Additionally, the relationship between breast volume over time and the following variables were analyzed: age, BMI, autologous technique, timing of reconstruction, indication of mastectomy, first postoperatively measured breast volume, ischemia time and irradiation status. Separate univariable models were built for each variable: $y=\beta_{0}+\beta_{1}x+\beta_{2}x^{2}+\beta_{3}x^{3}+\beta_{4}x^{4}+\beta_{5}v+\beta_{6}vx+\beta_{7}vx^{2}+\beta_{8}vx^{3}+\beta_{9}vx^{4}$ (model 2), where y is breast volume, $\beta_{0}$ is the intercept, $\beta_{1-9}$ are regression coefficients, v is one of the variables of interest and x is the standardized time postoperatively.

All models were fit using the statistical program R, Version 3.4.4. (R Foundation for Statistical Computing, Vienna, Austria). Supplementary material to the statistical analysis is included in Supplemental Digital Content. A value of p < 0.05 was considered statistically significant.

## 3 Results

**Fig. 2:**
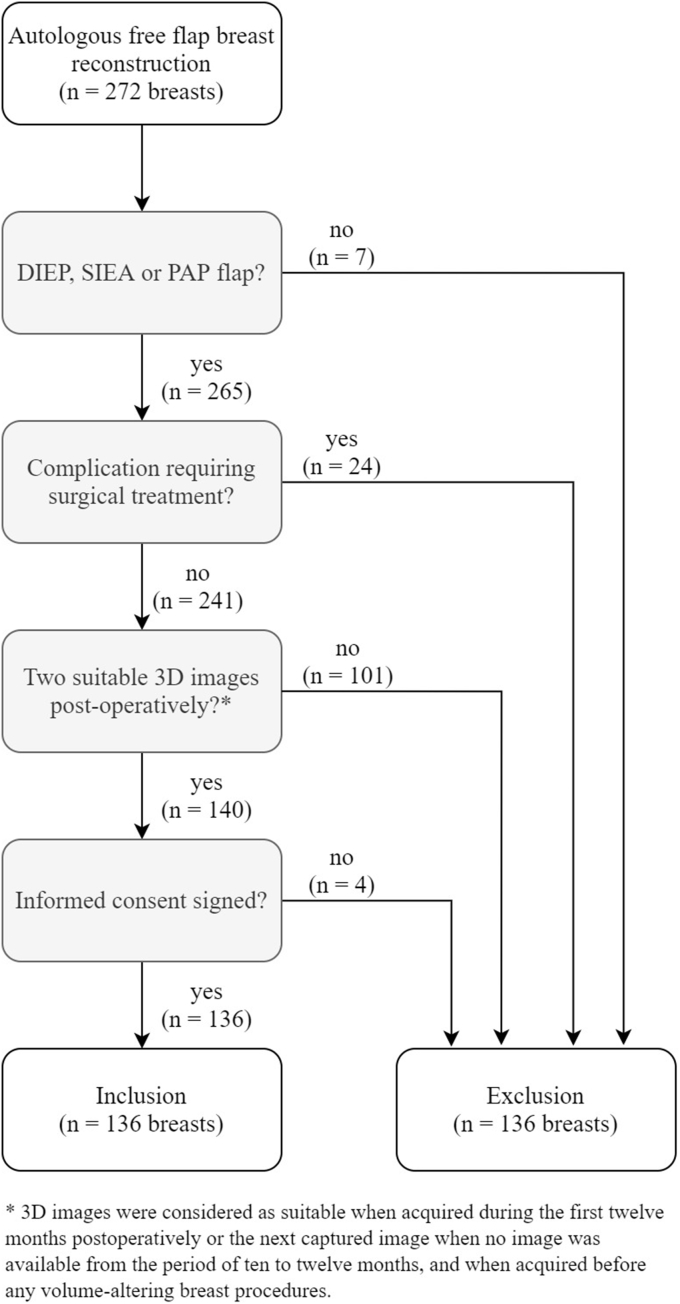
Flow chart of breast inclusion.

A total of 272 breasts in 191 patients were reconstructed with autologous tissue between December 2016 and January 2019. Based on the study criteria, a total of 136 breasts in 101 patients were included (Fig. 2). Patient and breast characteristics are shown in Table 1 and Table 2 respectively. The mean age of the patients was 49.1 years (range 26–70 years) and the mean body mass index (BMI) was 26.65 kg/m^2^ (range 21.45–31.96 kg/m^2^). Of these breasts, 126 were reconstructed with an abdominal-based free flap (DIEP n = 124, SIEA n = 2) and 10 with a PAP flap. Patients who underwent PAP flap reconstruction were statistically significant younger in age (mean 39.6 vs. 49.9 years, p = 0.007, 95% CI [2.9, 17.6]) and had a lower BMI (24.16 vs. 26.85 kg/m^2^, p = 0.002, 95% CI [0.98, 4.40]) compared to patients who underwent abdominal-based flap reconstruction.

**Table 1:** Patient characteristics at time of reconstruction.

Patient characteristic	Value	
Sample size; n	101	
Age; mean ± SD (years)	49.1	±10.4
BMI; mean ± SD (kg/m^2^)	26.65	±2.41
Laterality; n (%)		
Unilateral	57	(56.4%)
Bilateral	44^a^	(43.5%)
Smoking status; n (%)		
Nonsmoker	58	(57.4%)
Ex-smoker	40	(39.6%)
Current smoker^b^	3	(3.0%)
Systemic (neo)adjuvant treatment; n (%)		
No systemic (neo)adjuvant treatment	33	(32.6%)
Chemotherapy only	15	(14.9%)
Hormonal therapy only	3	(3.0%)
Chemotherapy and hormonal therapy	50	(49.5%)
High-risk genetic mutation; n (%)		
High-risk mutation^c^	28	(27.7%)
No mutation	25	(24.8%)
Unknown (not tested)	47	(46.5%)

a In 9 patients, one of two reconstructed breasts is excluded.

b Defined as actively smoking at the time of, quitting smoking <3 months prior to and/or restarting smoking after reconstruction.

c BRCA1, BRCA2 and CHEK2 mutation.

**Table 2:** Reconstructed breast characteristics.

Breast characteristic	Value	
Sample size; n	136	
Ischemia time; mean ± SD (minutes)^a^	58.9	±23.7
Breast side; n (%)		
Left	64	(47.1%)
Right	72	(52.9%)
Reconstruction type; n (%)		
DIEP	124	(91.1%)
SIEA	2	(1.5%)
PAP	10	(7.4%)
Timing; n (%)		
Primary	49	(36.0%)
Secondary	65	(47.8%)
Tertiary	22	(16.2%)
Reason for mastectomy; n		
In situ or invasive breast cancer	77	(56.6%)
Prophylactic	53	(39.0%)
Prophylactic after lumpectomy	6	(4.4%)
Radiation before reconstruction; n (%)	52	(38.2%)
Axillary lymph node dissection; n (%)	36	(26.5%)
Reconstruction combined with LNT	22	(16.2%)

LNT = lymph node transplantation.

a 126 breasts (in 10 cases ischemia time unknown).

### 3.1 Breast volume change over time

**Fig. 3:**
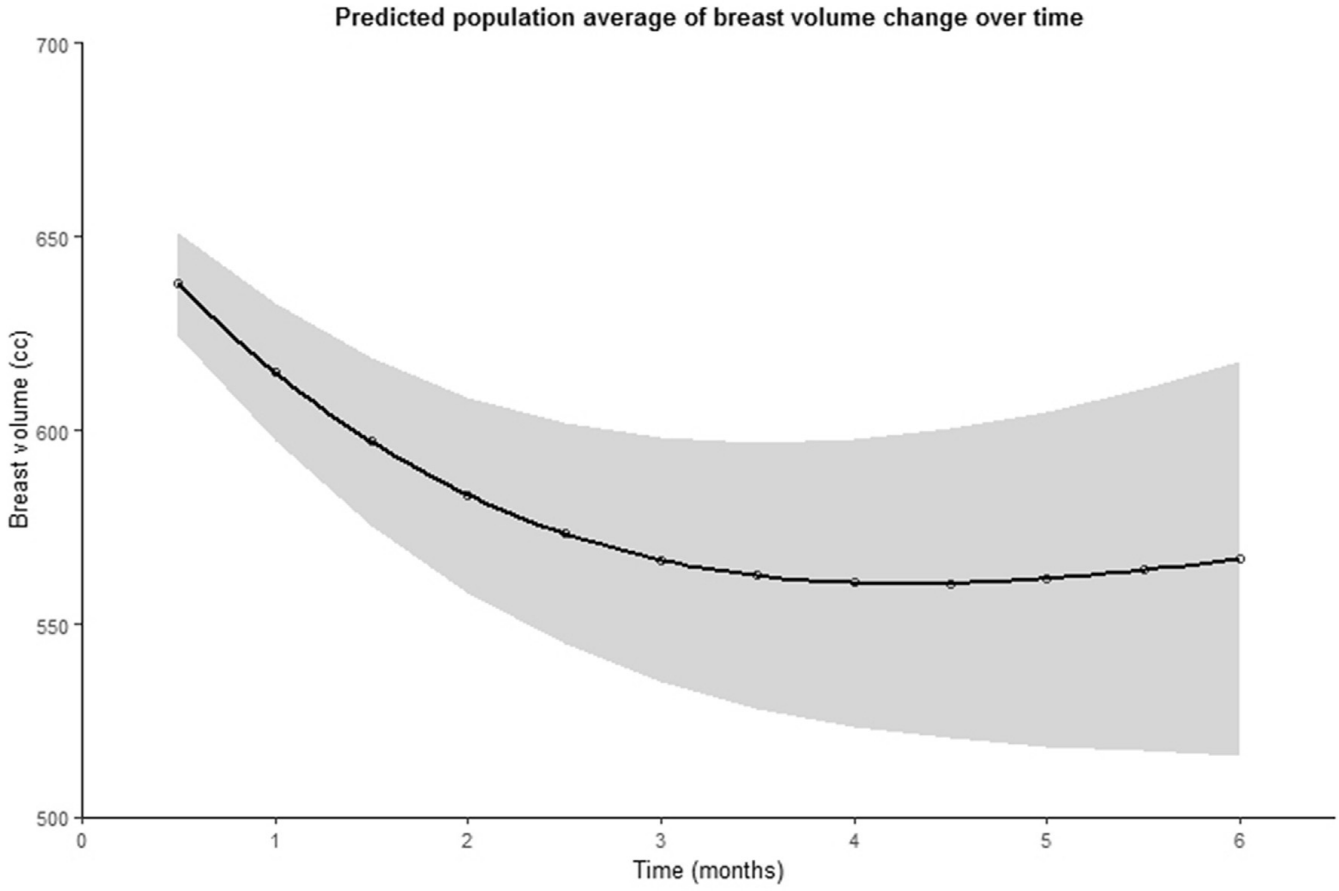
Predicted population average of breast volume change over six months (with 95% confidential interval) following autologous free flap reconstruction.

A total of 349 breast volume measurements of 136 breasts were obtained, with a mean time of 5.0 months postoperatively (±4.4 months, range 12 days–21.6 months). The postoperative breast volumes ranged from 167.9 to 1594.1 cc (mean 580.6 ± 220.6 cc). Using the nested mixed effects model, the predicted population average breast volume change over time was determined. The parameter estimates and the corresponding curve are shown in Table 3 and Fig. 3. From this data, it is apparent that the predicted volume of the breast decreased on average over time, with the course being characterized by an initial rapid breast volume decrease, followed by a slower decrease and then volume stabilization. As Fig. 3 shows, there was a statistically significant decrease in breast volume of 71.2 cc (11.2%) after three months and 71.1 cc (11.1%) after six months compared to two weeks postoperatively (mean breast volume 0.5 months: 637.8 cc, 95%-CI [624.4, 651.1]; 3 months: 566.6 cc, 95%-CI [535.1, 598.0]; 6 months: 567.6 cc, 95%-CI [515.9, 617.6]).

**Table 3:** Nested mixed effects model (model 1).

		β-value	LCI	UCI	p-value	
Intercept	$\beta_{0}$	561.803	518.290	605.315	<0.001	***
x	$\beta_{1}$	16.650	−12.882	46.181	0.268	
x^2^	$\beta_{2}$	37.617	21.529	53.706	<0.001	***
x^3^	$\beta_{3}$	−40.270	−60.876	−19.664	<0.001	***
x^4^	$\beta_{4}$	8.374	3.350	13.398	0.001	**

Standardized time variable (x) = .$\frac{time-mean}{standard\;deviation}$

LCI = lower confidential interval, UCI = upper confidential interval.

p-values <0.05 = *, p < 0.01 = ** and p < 0.001 = ***.

### 3.2 Factors associated with postoperative changes in breast volume

The parameter estimates ($\beta_{5}-\beta_{9}$) for each of the variables of interest using model 2 are shown in Table 4 (see Table A2, Supplemental Digital Content, which shows a complete overview with the coefficients $\beta_{0}-\beta_{4}$ per variable).

**Table 4:** Univariable nested mixed-effects model (model 2). Regression coefficients $\beta_{0-4}$ not shown; complete overview is included in Supplemental Digital Content.

Variable		β-value	LCI	UCI	p-value	
Age^a^	$\beta_{5}$	1.711	−2.543	5.964	0.427	
Age x	$\beta_{6}$	0.863	−2.230	3.957	0.583	
Age x^2^	$\beta_{7}$	−0.471	−1.936	0.995	0.527	
Age x^3^	$\beta_{8}$	0.346	−1.783	0.274	0.749	
Age x^4^	$\beta_{9}$	−0.098	−0.609	0.412	0.705	
BMI^a^	$\beta_{5}$	55.219	40.680	69.757	<0.001	***
BMI x	$\beta_{6}$	11.367	−0.639	23.372	0.063	
BMI x^2^	$\beta_{7}$	1.726	−4.877	8.330	0.607	
BMI x^3^	$\beta_{8}$	−4.472	−12.976	4.032	0.301	
BMI x^4^	$\beta_{9}$	0.717	−1.581	3.015	0.539	
Autologous technique^b^	$\beta_{5}$	−335.208	−506.741	−163.675	<0.001	***
Autologous technique x	$\beta_{6}$	−162.118	−340.667	16.432	0.075	
Autologous technique x^2^	$\beta_{7}$	27.099	−49.056	103.255	0.484	
Autologous technique x^3^	$\beta_{8}$	87.427	−34.411	209.264	0.159	
Autologous technique x^4^	$\beta_{9}$	−25.185	−63.414	13.045	0.196	
Timing^b^	$\beta_{5}$	−86.970	−164.185	−9.755	0.029	*
Timing x	$\beta_{6}$	29.211	−31.561	89.983	0.344	
Timing x^2^	$\beta_{7}$	−46.830	−79.294	−14.366	0.005	**
Timing x^3^	$\beta_{8}$	16.245	−25.994	58.484	0.449	
Timing x^4^	$\beta_{9}$	−1.963	−12.310	8.384	0.709	
Indication^b^	$\beta_{5}$	−25.945	−85.300	33.410	0.381	
Indication x	$\beta_{6}$	6.708	−51.688	65.104	0.821	
Indication x^2^	$\beta_{7}$	−55.334	−85.194	−21.473	0.001	**
Indication x^3^	$\beta_{8}$	33.606	−7.520	74.731	0.109	
Indication x^4^	$\beta_{9}$	−5.942	−16.097	4.212	0.250	
First PO breast volume^a^	$\beta_{5}$	0.845	0.763	0.926	<0.001	***
First PO breast volume x	$\beta_{6}$	0.118	−0.056	0.261	0.107	
First PO breast volume x^2^	$\beta_{7}$	0.127	0.0476	0.207	0.002	**
First PO breast volume x^3^	$\beta_{8}$	−0.120	−0.229	−0.010	0.032	*
First PO breast volume x^4^	$\beta_{9}$	0.019	−0.016	0.055	0.282	
Ischemia time^a^	$\beta_{5}$	−1.243	−2.848	0.362	0.125	
Ischemia time x	$\beta_{6}$	−0.445	−2.136	1.247	0.605	
Ischemia time x^2^	$\beta_{7}$	0.121	−0.754	0.996	0.786	
Ischemia time x^3^	$\beta_{8}$	0.324	−0.961	1.610	0.619	
Ischemia time x^4^	$\beta_{9}$	−0.173	−0.672	0.326	0.496	
Radiotherapy^b^	$\beta_{5}$	−35.544	−101.990	30.902	0.285	
Radiotherapy x	$\beta_{6}$	−8.644	−70.750	53.462	0.784	
Radiotherapy x^2^	$\beta_{7}$	−26.595	−59.237	6.047	0.110	
Radiotherapy x^3^	$\beta_{8}$	29.198	−14.742	73.139	0.192	
Radiotherapy x^4^	$\beta_{9}$	−6.823	−17.978	4.332	0.229	

Standardized time variable (x) = $\frac{time-mean}{standard\;deviation}$.

PO = postoperative, LCI = lower confidential interval, UCI = upper confidential interval.

p-values <0.05 = *, p < 0.01 = ** and p < 0.001 = ***.

a Continuous variables: age, BMI, first PO breast volume and ischemia time.

b Binary variables (0 vs. 1): autologous technique = abdominal vs. PAP flap; timing = primary vs. secondary + tertiary; indication = prophylactic vs. breast cancer, radiotherapy = no vs. yes.

**Fig. 4:**
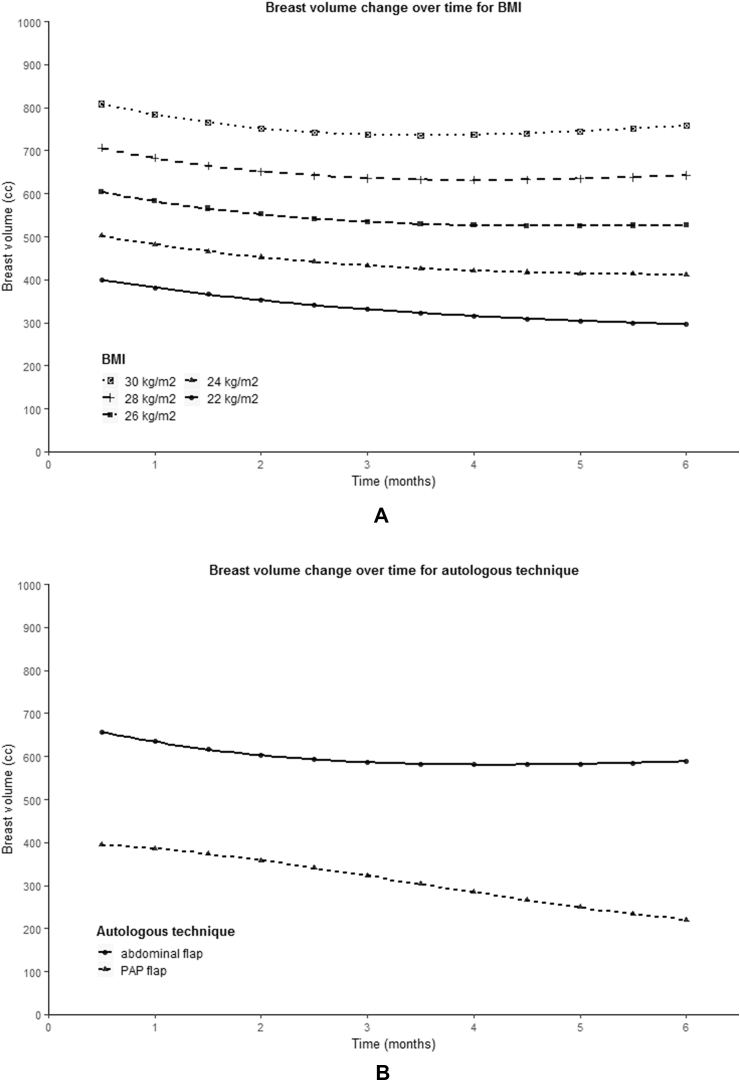

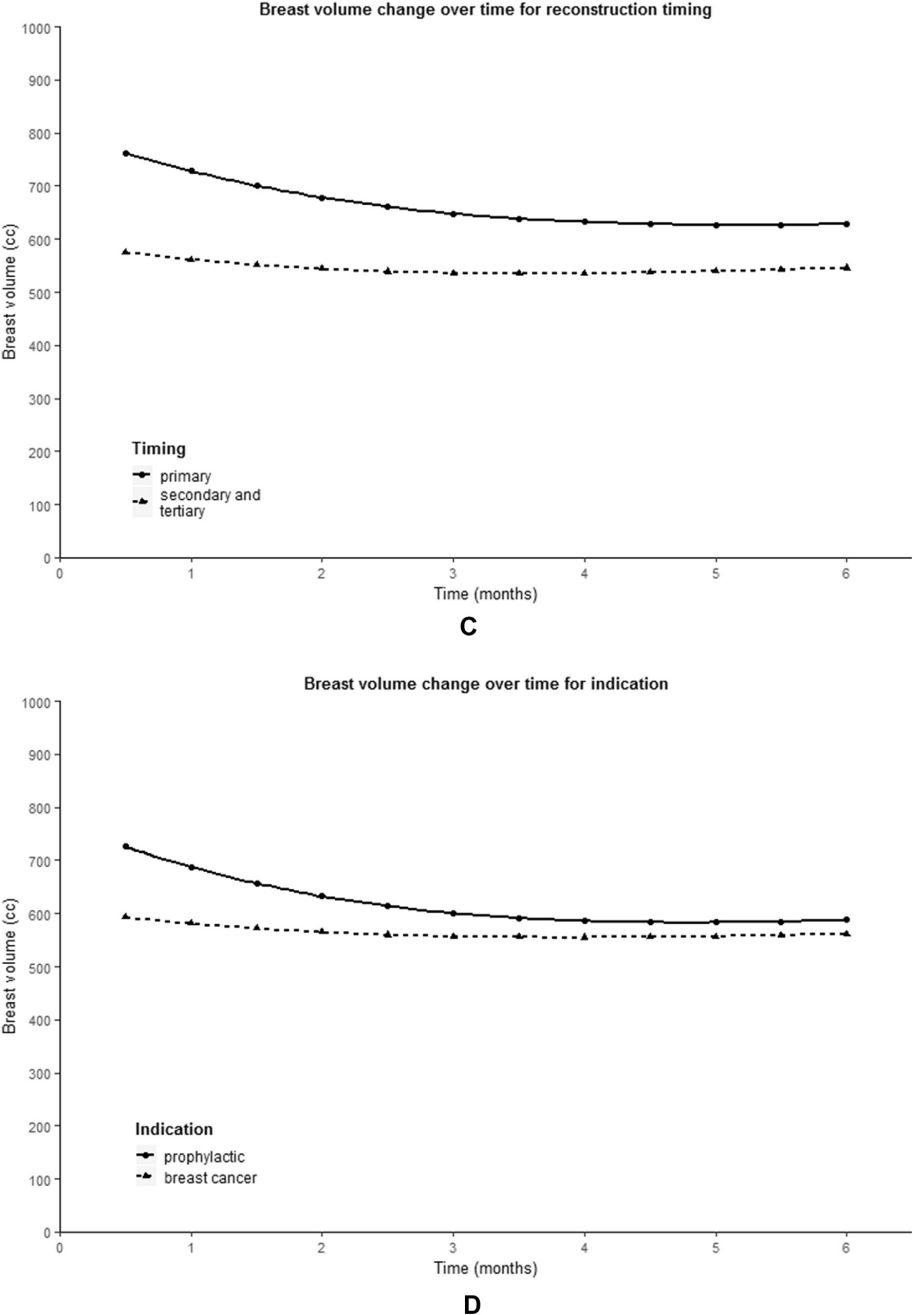

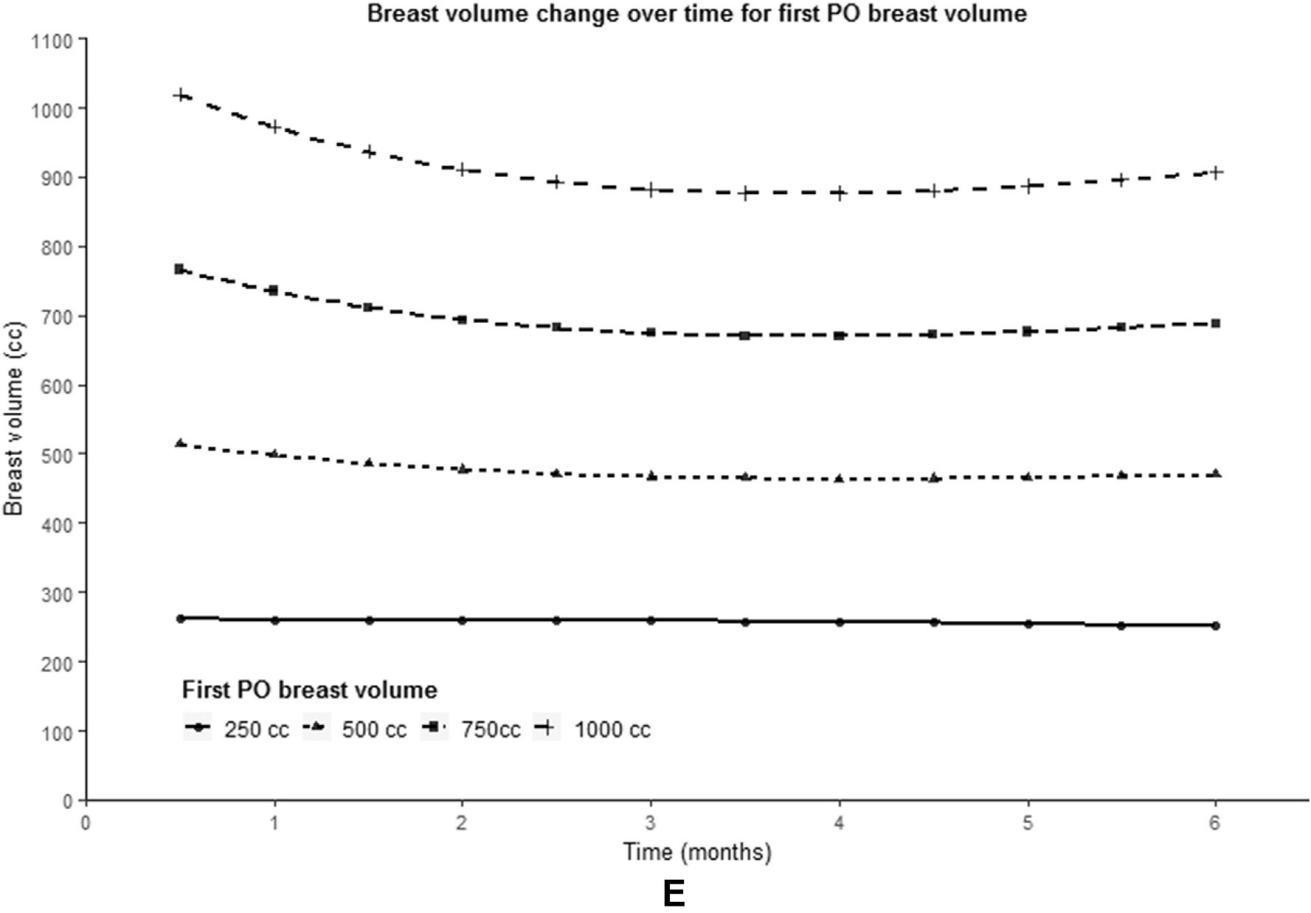
Predicted population average of breast volume change over six months for different variables. (A) BMI, (B) autologous technique, (C) reconstruction timing, (D) mastectomy indication, (E) first postoperatively (PO) measured breast volume.

The variables BMI (p < 0.001), autologous technique (p < 0.001), timing (p = 0.029) and first postoperatively measured breast volume (p < 0.001) showed a statistically significant difference in initial reconstructed breast volume (Fig. 4A, 4B, 4C, 4E). BMI and first postoperatively measured breast volume showed a positive correlation with the starting volume. PAP flap reconstruction showed to have a lower starting breast volume compared to abdominal-based flaps (mean 394.6 vs. 657.8 cc), as well as delayed and salvage breast reconstruction compared to primary reconstruction (mean 575.8 vs. 762.4 cc).

A statistically significant relationship between time and breast volume was observed for the variables timing ($\beta_{7}$ p = 0.005), mastectomy indication ($\beta_{7}$ p = 0.001) and first postoperatively measured breast volume ($\beta_{7}$ p = 0.002, $\beta_{8}$ p = 0.032) (Fig. 4C, 4D, 4E). As can be seen from the graphs presented in Fig. 4, a larger first postoperatively measured breast volume seemed to be associated with a larger decrease in breast volume. In addition, a prophylactic mastectomy appeared to result in a greater decrease in breast volume compared to a mastectomy in the context of breast cancer, just like primary reconstruction compared to secondary and tertiary breast reconstruction.

No statistically significant difference in starting breast volume and shape of the relationship between time and breast volume was found for the variables age, ischemia time and radiotherapy.

## 4 Discussion

This study investigated postoperative breast volume changes following autologous free perforator flap breast reconstruction using 3D imaging. An average decrease of predicted breast volume over time was found, with a mean predicted breast volume decrease of 11.2% after three and 11.1% after six months postoperatively. Reconstruction timing and first postoperatively measured breast volume showed a statistically significant difference in starting breast volume and in the shape of the relationship between time and breast volume, whereas autologous technique and BMI only showed a statistically significant difference in starting volume and mastectomy indication in the shape of the relationship.

Multiple studies have been conducted into the quantitative analysis of postoperative changes in flap volume in head and neck reconstruction [[7], [8], [9], [10]]. However, the flaps used in head and neck surgery are mainly free musculocutaneous flaps of which it is well-known that these flaps undergo volume reduction mainly due to muscle atrophy, whereas for breast reconstruction mainly free cutaneous flaps are used [15]. Despite this, several reports have studied changes in the size of transplanted fatty tissue in free flaps during the first postoperative year. Fujioka et al. [7] evaluated 19 postoperative microsurgical flaps over 6–10 months and reported an average postoperative fat thickness of 80.1% (range 60.6–99.7%), while Kimura et al. [8] described a decrease in fat volume to 75.0% at 1 year after surgery in 13 pedicled and 4 free myocutaneous flaps. In addition, Sakamoto et al. [10] reported an average final fat volume of 85.5% (±5.7%) at 12 months postoperatively in 21 free rectus abdominis myocutaneous flaps, with a slight increase of average fat volume from 6 to 12 months. These values show a more marked reduction compared to the predicted final breast volume of 88.9% after six months in this study. Differences in the volume reduction may be related to the differences in the patient population, used flaps and measurement techniques to evaluate flap volume. The mechanisms of fatty tissue atrophy are not well clarified yet, but the decrease in flap volume may partly be explained by early postoperative factors, such as apoptosis [16] or improvement of postoperative edema and inflammation, but other factors such as flap denervation and ischemic changes due to transient ischemia may also play a role. In this study, two weeks postoperatively was taken as the baseline measurement, since it is expected that most postoperative swelling would have decreased, making it a more reliable measurement moment compared to a few days postoperatively.

To date, there is no literature available on factors that influence initial reconstructed breast volume after autologous reconstruction. In the presented study, initial BMI, autologous technique, reconstruction timing and first postoperatively measured breast volume showed to significantly contribute to a difference in initial reconstructed breast volume. The positive correlation between initial reconstructed breast volume and BMI is likely to be related to an increased subcutaneous fat tissue in patients with a higher BMI and therefore the possibility of harvesting larger flap volumes, as well a higher volume of the original breast that is being pursued. A possible explanation for the difference in initial reconstructed volume between abdominal-based and PAP flap reconstruction is the significant difference in BMI between the two groups since the two curves appear to match the curves for the corresponding mean BMI (Fig. 4). However, also the differences between the two flaps and their donor sites are likely to contribute to the difference in initial reconstructed volume since often higher flap volumes can be harvested abdominally compared to the inner thigh. The higher initial reconstructed breast volume after primary reconstruction compared to secondary and tertiary reconstruction might be explained by the difference in breast preparation and flap in setting, whereby in the case of primary reconstruction a skin-sparing mastectomy is often performed with preservation of the skin-envelope, whereas with secondary reconstruction the lower mastectomy skin is usually excided and the inferior edge of the flap is used to recreate the inframammary fold.

Among the factors examined in the present study, reconstruction timing, mastectomy indication and first postoperatively measured breast volume showed a significant association with flap volume changes over time. Other studies on the association between factors and postoperative fat volume changes in flaps are scarce, often only examining the effect of postoperative radiotherapy. Preoperative irradiation, however, showed no association with postoperative changes in flap volume, which is consistent with the results of the study by Kimura et al. [8] Yamaguchi et al. [9] suggested that the fat volume changes in flaps are influenced by host conditions. This is supported by the same study of Kimura et al. [8] which showed a significant association between weight loss and the reduction in total flap volume. Unfortunately, we were unable to correct for possible weight changes because the BMI was not systematically collected at each time point. Ultimately, although ischemia time is associated with fat necrosis, no significant difference was found in the shape of the relationship between time and breast volume [17].

Over the years, various studies have been conducted to validate 3D surface imaging (3D-SI) systems for measuring breast volumes [[18], [19], [20], [21], [22], [23], [24], [25], [26]]. A literature review by O’Connell et al. stated that the reproducibility of 3D-SI is of crucial importance in studies of evolving changes over time, as clinically relevant findings can be masked or exaggerated by small variations in patient positioning, placement of landmarks and intra- or inter-observer measurements [27]. In our research, all images were acquired by the same photographer with the patients in the same positioning. Furthermore, inter-observer bias was minimized because only one examiner measured all breast volumes. Intra-observer bias, on the other hand, continues to exist because only one measurement was made. However, another study by O’Connell et al. [25] validating the VECTRA XT in a breast cancer population showed a low intra-observer variation (coefficient of variation <4%).

The intention of this study was to analyze breast volume changes over twelve months. However, as many patients underwent correction procedures of the breast after six months, it was decided to only evaluate the period up to six months postoperatively to keep the results pure. Secondly, when the data was collected, it was discovered that the used 3D images showed a large spread in the number and timing of the measurement moments between patients due to logistical reasons. This required a more complex statistical analysis than had been estimated in advance. The nested mixed effects model derived a predicted population average of breast volume changes over time, whereby due to the large spread in breast volume and time course, the findings cannot be extrapolated to every patient. However, the model overcomes the shortcomings of traditional analysis, as it accounts for the correlation between time points (without collapsing longitudinal data into time intervals and ignoring data from specific time points) and within-patient breast volume changes for each separate breast.

The present study lays the groundwork for further research into postoperative breast volume changes following autologous reconstruction. Further prospective research should be undertaken on flap volume changes and the identification of factors that have an influence on this, to ideally create a prediction model for plastic surgeons to facilitate predictable surgical outcomes. Nevertheless, the findings of this study are the first to show that breasts undergo volume changes, which is something to be aware of in daily practice.

## 5 Conclusion

This study is the first to evaluate early flap volumes changes following autologous free breast reconstruction and the factors influencing this. The predicted final flap volumes decreased overall to 88.9% of its original volume after six months. A natural progression of this work is to analyze whether a 12.5% overcorrection of flap volume used for autologous reconstruction confirms this conclusion. Additionally, the results of this study show that specific factors can influence the final flap volume, which emphasizes the importance of gaining more insight into these factors.

## Declaration of competing interest

The authors declare that there is no conflict of interest.
