# Supplementary material for: “Three-dimensional evaluation of breast volume changes following autologous free flap breast reconstruction over six months”
Source: Breast. 2020 Feb 10;50:85–94. doi: 10.1016/j.breast.2020.02.005 (PMC7380344; doi:10.1016/j.breast.2020.02.005)
Supplement: Multimedia component 1 [file mmc1.docx]

**Supplemental Digital Content: Supplementary material to statistical analysis**

*1. Determination of polynomial degree*

A likelihood ratio test comparing the Akaike Information Criteria (AIC) was run, to determine which of the polynomial functions was the best fit (Table A1). The likelihood ratio test between the fourth- and fifth-degree polynomial showed a statistically significant difference with very similar AIC’s. Therefore, the fourth-degree polynomial was chosen to prevent the curve from overfitting.

**Table A1 Goodness-of-fit and likelihood-ratio test for polynomial function**

| Polynomial degree | AIC | Likelihood-ratio test comparing AIC | | | |
| --- | --- | --- | --- | --- | --- |
|  |  | **First degree** | **Second degree** | **Third degree** | **Fourth degree** |
| First degree | 4437 | -- | -- | -- | -- |
| Second degree | 4422 | <0.0001 | -- | -- | -- |
| Third degree | 4414 | <0.0001 | 0.0015 | -- | -- |
| Fourth degree | 4401 | <0.0001 | <0.0001 | 0.0016 | -- |
| Fifth degree | 4400 | <0.0001 | <0.0001 | <0.0001 | 0.0603 |
| AIC = Akaike Information Criterion | | | | | |

*2. Univariable nested mixed-effects model*

The relationship between breast volume over time and the variables of interest were analyzed by building separate univariable, nested mixed-effects models for each variable, with the variable of interest added as an additional fixed effect, by multiplying the variable with the quartic polynomial, allowing each value of each variable to have its own intercept and slope:

$y=\beta_{0}+\beta_{1}x+{\beta_{2}x}^{2}+\beta_{3}x^{3}+{\beta_{4}x}^{4}+\beta_{5}v+\beta_{6}vx+{\beta_{7}vx}^{2}+\beta_{8}{vx}^{3}+{\beta_{9}vx}^{4}$ (model 2)

Where y is the breast volume, $\beta_{0}$ is the intercept, $\beta_{1-9}$ are regression coefficients, v is the variable of interest and x is the standardized time. The parameter estimates of the separate variable models using model 2 are shown in Table A2.

**Table A2 Univariable nested mixed-effects model (model 2)**

| Variable |  | β-value | LCI | UCI | p-value |  |
| --- | --- | --- | --- | --- | --- | --- |
| Age ^a^ |  |  |  |  |  |  |
| Intercept | $\beta_{0}$ | 578.0.0 | 267.424 | 689.039 | <0.001 | *** |
| x | $\beta_{1}$ | -28.456 | -170.508 | 113.596 | 0.693 |  |
| x^2^ | $\beta_{2}$ | 61.058 | -10.586 | 132.541 | 0.094 |  |
| x^3^ | $\beta_{3}$ | -53.219 | -149.625 | 43.188 | 0.278 |  |
| x^4^ | $\beta_{4}$ | 11.864 | -10.586 | 34.313 | 0.299 |  |
| Age | $\beta_{5}$ | 1.711 | -2.543 | 5.964 | 0.427 |  |
| Age $\boldsymbol{\cdot}$ x | $\beta_{6}$ | 0.863 | -2.230 | 3.957 | 0.583 |  |
| Age $\boldsymbol{\cdot}$ x^2^ | $\beta_{7}$ | -0.471 | -1.936 | 0.995 | 0.527 |  |
| Age $\boldsymbol{\cdot}$ x^3^ | $\beta_{8}$ | 0.346 | -1.783 | 0.274 | 0.749 |  |
| Age $\boldsymbol{\cdot}$ x^4^ | $\beta_{9}$ | -0.098 | -0.609 | 0.412 | 0.705 |  |
| BMI ^a^ |  |  |  |  |  |  |
| Intercept | $\beta_{0}$ | -910.892 | -1297.481 | -524.303 | <0.001 | *** |
| x | $\beta_{1}$ | -290.804 | -612.206 | 30.598 | 0.076 |  |
| x^2^ | $\beta_{2}$ | -8.829 | -184.539 | 166.881 | 0.921 |  |
| x^3^ | $\beta_{3}$ | 81.341 | -144.822 | 307.504 | 0.479 |  |
| x^4^ | $\beta_{4}$ | -11.312 | -72.286 | 49.662 | 0.715 |  |
| BMI | $\beta_{5}$ | 55.219 | 40.680 | 69.757 | <0.001 | *** |
| BMI $\boldsymbol{\cdot}$ x | $\beta_{6}$ | 11.367 | -0.639 | 23.372 | 0.063 |  |
| BMI $\boldsymbol{\cdot}$ x^2^ | $\beta_{7}$ | 1.726 | -4.877 | 8.330 | 0.607 |  |
| BMI$\boldsymbol{\cdot}$ x^3^ | $\beta_{8}$ | -4.472 | -12.976 | 4.032 | 0.301 |  |
| BMI$\boldsymbol{\cdot}$ x^4^ | $\beta_{9}$ | 0.717 | -1.581 | 3.015 | 0.539 |  |
| Autologous technique ^b^ |  | |  |  |  |  |
| Intercept | $\beta_{0}$ | 582.714 | 539.721 | 625.706 | <0.001 | *** |
| x | $\beta_{1}$ | 19.694 | -10.737 | 50.125 | 0.203 |  |
| x^2^ | $\beta_{2}$ | 37.718 | 21.008 | 54.428 | <0.001 | *** |
| x^3^ | $\beta_{3}$ | -41.901 | -63.405 | -20.397 | <0.001 | *** |
| x^4^ | $\beta_{4}$ | 8.735 | 3.544 | 13.926 | 0.001 | ** |
| Autologous technique | $\beta_{5}$ | -335.208 | -506.741 | -163.675 | <0.001 | *** |
| Autologous technique $\boldsymbol{\cdot}$ x | $\beta_{6}$ | -162.118 | -340.667 | 16.432 | 0.075 |  |
| Autologous technique $\boldsymbol{\cdot}$ x^2^ | $\beta_{7}$ | 27.099 | -49.056 | 103.255 | 0.484 |  |
| Autologous technique $\boldsymbol{\cdot}$ x^3^ | $\beta_{8}$ | 87.427 | -34.411 | 209.264 | 0.159 |  |
| Autologous technique $\boldsymbol{\cdot}$ x^4^ | $\beta_{9}$ | -25.185 | -63.414 | 13.045 | 0.196 |  |
| Timing ^b^ |  |  |  |  |  |  |
| Intercept | $\beta_{0}$ | 626.798 | 588.758 | 694.839 | <0.001 | *** |
| x | $\beta_{1}$ | -4.716 | -51.464 | 42.032 | 0.843 |  |
| x^2^ | $\beta_{2}$ | 65.672 | 40.028 | 91.317 | <0.001 | *** |
| x^3^ | $\beta_{3}$ | -47.143 | -78.462 | -15.825 | 0.003 | ** |
| x^4^ | $\beta_{4}$ | 8.753 | 1.604 | 15.902 | 0.017 | * |
| Timing | $\beta_{5}$ | -86.970 | -164.185 | -9.755 | 0.029 | * |
| Timing$\boldsymbol{\cdot}$ x | $\beta_{6}$ | 29.211 | -31.561 | 89.983 | 0.344 |  |
| Timing$\boldsymbol{\cdot}$ x^2^ | $\beta_{7}$ | -46.830 | -79.294 | -14.366 | 0.005 | ** |
| Timing$\boldsymbol{\cdot}$ x^3^ | $\beta_{8}$ | 16.245 | -25.994 | 58.484 | 0.449 |  |
| Timing$\boldsymbol{\cdot}$ x^4^ | $\beta_{9}$ | -1.963 | -12.310 | 8.384 | 0.709 |  |
| Indication ^b^ |  |  |  |  |  |  |
| Intercept | $\beta_{0}$ | 583.267 | 524.286 | 642.249 | <0.001 | *** |
| x | $\beta_{1}$ | 8.877 | -33.585 | 51.338 | 0.681 |  |
| x^2^ | $\beta_{2}$ | 81.470 | 46.338 | 96.601 | <0.001 | *** |
| x^3^ | $\beta_{3}$ | -58.356 | -57.747 | -28.963 | <0.001 | *** |
| x^4^ | $\beta_{4}$ | 11.153 | 4.334 | 17.971 | 0.002 | ** |
| Indication | $\beta_{5}$ | -25.945 | -85.300 | 33.410 | 0.381 |  |
| Indication$\boldsymbol{\cdot}$ x | $\beta_{6}$ | 6.708 | -51.688 | 65.104 | 0.821 |  |
| Indication$\boldsymbol{\cdot}$ x^2^ | $\beta_{7}$ | -55.334 | -85.194 | -21.473 | 0.001 | ** |
| Indication$\boldsymbol{\cdot}$ x^3^ | $\beta_{8}$ | 33.606 | -7.520 | 74.731 | 0.109 |  |
| Indication$\boldsymbol{\cdot}$ x^4^ | $\beta_{9}$ | -5.942 | -16.097 | 4.212 | 0.250 |  |
| First PO breast volume ^a^ | |  |  |  |  |  |
| Intercept | $\beta_{0}$ | 43.077 | -8.201 | 94.355 | 0.099 |  |
| x | $\beta_{1}$ | -43.684 | -131.087 | 43.720 | 0.326 |  |
| x^2^ | $\beta_{2}$ | -41.397 | -90.656 | 7.863 | 0.099 |  |
| x^3^ | $\beta_{3}$ | 29.708 | -35.914 | 95.329 | 0.373 |  |
| x^4^ | $\beta_{4}$ | -30.23 | -22.748 | 16.701 | 0.763 |  |
| First PO breast volume | $\beta_{5}$ | 0.845 | 0.763 | 0.926 | <0.001 | *** |
| First PO breast volume $\boldsymbol{\cdot}$ x | $\beta_{6}$ | 0.118 | -0.056 | 0.261 | 0.107 |  |
| First PO breast volume $\boldsymbol{\cdot}$ x^2^ | $\beta_{7}$ | 0.127 | 0.0476 | 0.207 | 0.002 | ** |
| First PO breast volume $\boldsymbol{\cdot}$ x^3^ | $\beta_{8}$ | -0.120 | -0.229 | -0.010 | 0.032 | * |
| First PO breast volume $\boldsymbol{\cdot}$ x^4^ | $\beta_{9}$ | 0.019 | -0.016 | 0.055 | 0.282 |  |
| Ischemia time ^a^ |  |  |  |  |  |  |
| Intercept | $\beta_{0}$ | 637.156 | 532.625 | 741.687 | <0.001 | *** |
| x | $\beta_{1}$ | 41.572 | -60.420 | 143.564 | 0.422 |  |
| x^2^ | $\beta_{2}$ | 30.236 | -23.967 | 84.438 | 0.273 |  |
| x^3^ | $\beta_{3}$ | -54.173 | -130.529 | 22.182 | 0.163 |  |
| x^4^ | $\beta_{4}$ | 16.395 | -11.568 | 44.358 | 0.249 |  |
| Ischemia time | $\beta_{5}$ | -1.243 | -2.848 | 0.362 | 0.125 |  |
| Ischemia time$\boldsymbol{\cdot}$ x | $\beta_{6}$ | -0.445 | -2.136 | 1.247 | 0.605 |  |
| Ischemia time$\boldsymbol{\cdot}$ x^2^ | $\beta_{7}$ | 0.121 | -0.754 | 0.996 | 0.786 |  |
| Ischemia time$\boldsymbol{\cdot}$ x^3^ | $\beta_{8}$ | 0.324 | -0.961 | 1.610 | 0.619 |  |
| Ischemia time$\boldsymbol{\cdot}$ x^4^ | $\beta_{9}$ | -0.173 | -0.672 | 0.326 | 0.496 |  |
| Radiotherapy ^b^ |  |  |  |  |  |  |
| Intercept | $\beta_{0}$ | 578.810 | 526.669 | 630.950 | <0.001 | *** |
| x | $\beta_{1}$ | 17.710 | -18.971 | 54.392 | 0.342 |  |
| x^2^ | $\beta_{2}$ | 48.049 | 27.688 | 68.411 | <0.001 | *** |
| x^3^ | $\beta_{3}$ | -48.782 | -74.509 | -23.055 | <0.001 | *** |
| x^4^ | $\beta_{4}$ | 10.068 | 3.941 | 16.194 | 0.001 | ** |
| Radiotherapy | $\beta_{5}$ | -35.544 | -101.990 | 30.902 | 0.285 |  |
| Radiotherapy$\boldsymbol{\cdot}$ x | $\beta_{6}$ | -8.644 | -70.750 | 53.462 | 0.784 |  |
| Radiotherapy$\boldsymbol{\cdot}$ x^2^ | $\beta_{7}$ | -26.595 | -59.237 | 6.047 | 0.110 |  |
| Radiotherapy$\boldsymbol{\cdot}$ x^3^ | $\beta_{8}$ | 29.198 | -14.742 | 73.139 | 0.192 |  |
| Radiotherapy$\boldsymbol{\cdot}$ x^4^ | $\beta_{9}$ | -6.823 | -17.978 | 4.332 | 0.229 |  |
| ^a^ Continuous variables: age, BMI, first PO breast volume and ischemia time.  ^b^ Binary variables (0 vs. 1): autologous technique = abdominal vs. PAP flap; timing = primary vs. secondary + tertiary; indication = prophylactic vs. breast cancer, radiotherapy = no vs. yes.  standardized time variable (x) = $\frac{\boldsymbol{time-mean}}{\boldsymbol{standard deviation}}$.  PO=postoperative, LCI = lower confidential interval, UCI = upper confidential interval.  p-values <0.05 = *, p<0.01 = ** and p<0.001 = ***. | | | | | | |
